# Persistent perineal morbidity is common following abdominoperineal excision for rectal cancer

**DOI:** 10.1007/s00384-015-2328-1

**Published:** 2015-08-06

**Authors:** Dan Asplund, Mattias Prytz, David Bock, Eva Haglind, Eva Angenete

**Affiliations:** Department of Surgery, Institute of Clinical Sciences, Sahlgrenska Academy, University of Gothenburg, SSORG - Scandinavian Surgical Outcomes Research Group, Sahlgrenska University Hospital/Östra, 416 85 Gothenburg, Sweden; Department of Surgery, NU Hospital group, Trollhättan, Sweden

**Keywords:** Rectal cancer, Abdominoperineal excision, Perineal morbidity, Quality of life

## Abstract

**Purpose:**

Short-term complications related to the perineal wound after abdominoperineal excision (APE) are a well-known problem. Perineal morbidity in the longer term is an almost unexplored area. The aim of this cross-sectional study was to investigate the prevalence of perineal symptoms 3 years after APE for rectal cancer, to identify potential risk factors and to explore the relationship between perineal morbidity and global quality of life.

**Method:**

All patients who underwent APE in Sweden between 2007 and 2009 (*n* = 1373) were identified through the Swedish Colorectal Cancer Registry. Surviving patients were contacted 3 years after surgery and asked about participation. A total of 545 patients completed a detailed questionnaire. Clinical data was collected from the registry and surgical charts.

**Results:**

Perineal symptoms occurred in 50 % of all patients 3 years after APE and more frequently in women (58 vs. 44 %; *p* = 0.001). Delayed healing of the perineal wound (>4 weeks) occurred in 25 % of all patients and more frequently after extralevator APE (ELAPE) than after conventional APE (32 vs. 11 %, *p* < 0.001). Delayed healing was associated with an increased risk of more severe perineal symptoms (relative risk (RR) 1.50, 95 % confidence interval (95 % CI) 1.09–2.05). Patients with more severe perineal symptoms (*n* = 129) had a significantly lower global quality of life as measured by EQ-5D visual analogue scale (VAS; median 75 vs. 83 points on the 100-point scale; *p* < 0.001).

**Conclusion:**

Persistent perineal symptoms are common after APE and may have an impact on patients’ quality of life. Delayed wound healing may be a risk factor for persistent symptoms. Further studies are needed to identify avoidable clinical factors for the development of persistent perineal morbidity.

**ClinicalTrials.gov Identifier:**

NCT01296984

## Introduction

Treatment of patients with distal rectal cancer is a clinical challenge. Achieving an optimal oncologic result is paramount, but functional consequences of treatment must also be considered. Recently, several studies have focused on the proposed oncological advantage of extralevator abdominoperineal excision (ELAPE) compared to conventional abdominoperineal excision (conventional APE) [2, 7, 17, 18, 24, 26]. Other studies have investigated functional aspects of these procedures such as sexual and urological dysfunction [6, 9] and stoma-related problems [1, 14].

The occurrence and implications of perineal symptoms following abdominoperineal excision is a largely unexplored area. One study found persistent perineal pain in half of all patients 2 years after ELAPE [23]. Reports of other long-term perineal symptoms are scarce. In contrast, short-term perineal morbidity after abdominoperineal excision is well documented. Several studies have reported increased perineal wound complications after ELAPE compared to conventional APE [2, 7, 11, 13, 18, 24, 25]. Perineal wound complications frequently result in delayed healing, but whether this is associated with persistent perineal symptoms is not known.

We hypothesized that persistent perineal symptoms are frequent following any kind of abdominoperineal excision. In the present analysis, we explored the prevalence of perineal symptoms about 3 years after surgery in a national cohort. The primary objective was to determine the prevalence and severity of symptoms, and secondary objectives were to identify risk factors for long-term perineal morbidity and to investigate the impact of perineal morbidity on patients’ health-related quality of life. Oncologic results in this national cohort, including 3-year recurrence rates, have been reported previously [17–19].

## Methods

In this registry-based, observational study, patients operated with abdominoperineal excision for rectal cancer in Sweden in the years 2007–2009 were identified through the Swedish Colorectal Cancer Registry. This national quality registry covers more than 97 % of all patients in Sweden and has a good internal validity [8, 15]. Data on sex, age, BMI, American Society of Anesthesiologists (ASA) classification, tumor height (distance from the lower edge of the tumor to the anal verge), neoadjuvant treatment, circumferential resection margin (CRM) involvement, and tumor pT and pN stage were retrieved from the registry. Data regarding perineal dissection and reconstruction techniques were not included in the registry but was extracted from operative notes [19]. This allowed for the classification of procedures as either conventional APE or ELAPE in a majority of cases, but surgical technique remained interminable in as many as 252 cases. Information regarding perineal symptoms after surgery was obtained through a study-specific questionnaire.

### Questionnaire

**Fig. 1:**
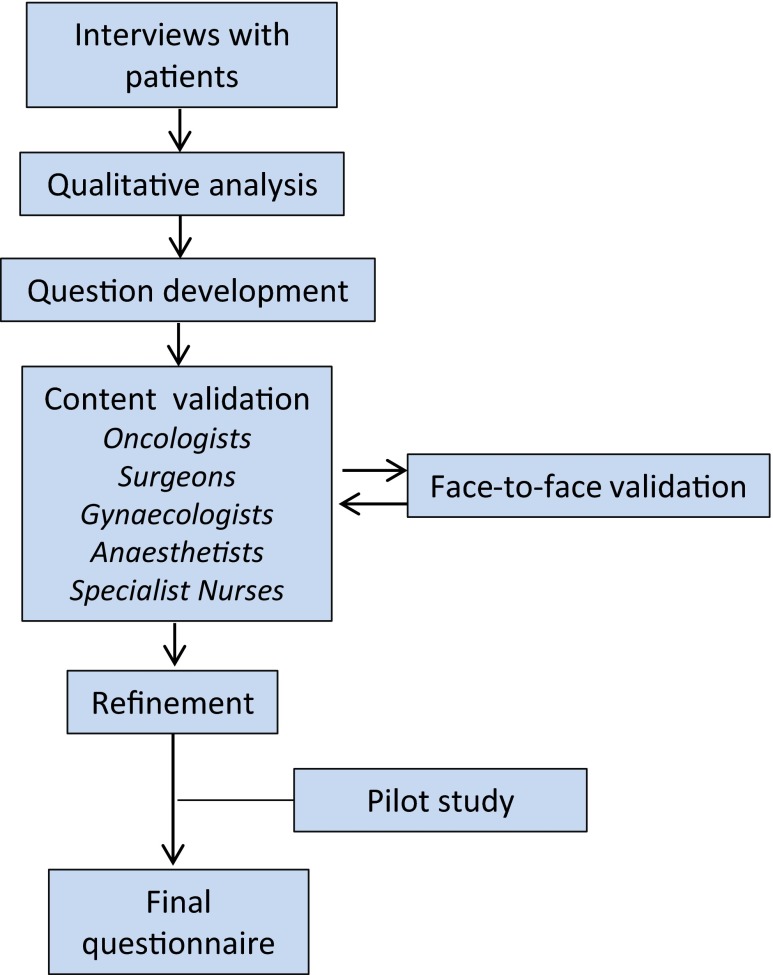
Development of a study-specific questionnaire

The questions on perineal symptoms (Table 1) analyzed in the present study were part of an extensive questionnaire that also covered many other aspects of functional outcome after abdominoperineal excision. The development and validation of this questionnaire is illustrated in Fig. 1 and has been described in detail elsewhere [3, 10, 12, 21, 22]. The process involved interviews with rectal cancer patients and subsequent analysis with qualitative methods, content validation in a multidisciplinary group of professionals with extensive clinical experience in the field, and face-to-face validation, where patients were asked to complete the questionnaire in the presence of a specialist nurse to detect any problems, misinterpretations, or concerns. Questions were revised accordingly, and the process continued until no uncertainties remained.

**Table 1:** Questions on perineal symptoms and perineal wound healing

Perineal symptoms
Have you had pain between the buttocks in the past month?
Have you had difficulties to sit in the past month?
Have you had loss of sensation/numbness in the buttocks in the past month?
Have you experienced tension in the buttocks in the past month?
Have you experienced a tingling/stinging sensation in the buttocks in the past month?
Have you experienced cramps/urgency that you perceived came from the previous location of your rectum in the past month?
Response options: Not at all/a little/quite a bit/very much
Perineal wound healing
For how long after your rectal surgery did you need to irrigate or change the dressing of the wound between the buttocks, at home or at a primary care centre/hospital?
Response options: 2 weeks/3–4 weeks/1–2 months/3–4 months/longer than 4 months
What was your experience of the wound healing process after your rectal surgery, when the wound between the buttocks was healing?
Response options: Not at all difficult/a little difficult/quite difficult/very difficult
Questions on symptom-associated distress (exemplified by the question regarding pain)
If you, for the rest of your life, would have as much pain between the buttocks as you did in the past month, would that distress you?
Response options: Not applicable, I have not had pain between the buttocks in the past month/not at all/a little/quite a bit/very much

During the development of the questionnaire, six “core” perineal symptoms emerged and were included in this analysis: pain, sitting disability, paraesthesia, tension between the buttocks, sensation of tingling/stinging between the buttocks, and perineal cramps/sensation of urgency. The recall period was the past month. Response options were dichotomized as explained in the tables. Patients reporting at least one perineal symptom of severe intensity (response options *quite a bit* or *very much*, see Table 1) were defined by us to have a *severe perineal morbidity*.

Also included in this analysis were questions on post-operative perineal wound healing and the EQ-5D visual analogue scale (VAS) question on global health-related quality of life [4, 16, 20].

### Statistical analyses

All data were collected in a database, and statistical analyses were performed using SPSS 21.0 (IBM SPSS Inc. Armonk, NY, USA) and SAS v. 9 (SAS institute). In the analysis of risk factors for severe perineal morbidity (the experience of one or more symptom of severe intensity, see above), variables chosen as potential predictors were radiotherapy, sex, age, tumor height, and delayed perineal wound healing. Because of the observed strong association between surgical technique and perineal wound healing (Table 2), surgical technique was not included in the regression model. In the subgroup of patients who underwent ELAPE, perineal repair and excision of the coccyx were included as potential predictors as well. A log-linear binomial regression model [5] was used to estimate the relative risk (RR) and 95 % confidence intervals (95 % CIs). In case the binomial model did not converge, a log-linear Poisson regression with a robust error variance was used [27]. Additional statistical analyses involved chi-square test and Mann–Whitney *U* test for categorical and continuous variables, respectively. No correction for multiple testing was made, and results should therefore be regarded as interesting findings rather than as conclusive evidence.

**Table 2:** Time to healing of the perineal wound stratified on surgical technique

			Conventional APE	ELAPE	*p* value	Missing
Time to healing (%)	Normal	0–4 weeks	62 (88.6 %)	146 (68.2 %)	0.001^a^	9
	Delayed	1–4 months	6 (8.6 %)	48 (22.4 %)		
		>4 months	2 (2.9 %)	20 (9.3 %)		

Patients operated with an indeterminate surgical technique (*n* = 252) are omitted

*APE* abdominoperineal excision, *ELAPE* extralevator abdominoperineal excision

^a^ *normal* versus *delayed* (>4 weeks) healing

## Results

**Fig. 2:**
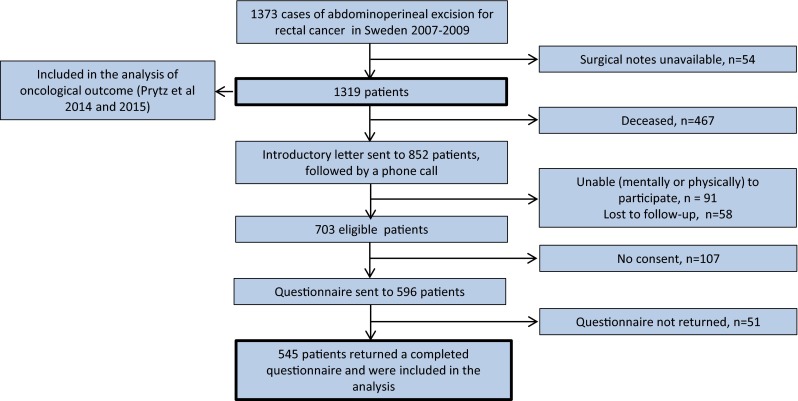
Flowchart of patients

Out of the 1319 patients who were included in our previous analyses of oncological outcome [17, 18], 852 were alive 3 years post-operatively and 703 patients were eligible for inclusion in this study (Fig. 2). A total of 596 patients agreed to receive the questionnaire by mail and 545 returned the questionnaire and were included in the analysis. Reasons for non-inclusion (*n* = 774) are presented in Fig. 2. Clinical characteristics of included patients, non-responders, and deceased patients are presented in Table 3. Non-responders were older and had more comorbidity as reflected by the preoperative ASA grade and received less neoadjuvant radiotherapy than responders to the questionnaire. Conventional APE was more common among non-responders, as were involved resection margins.

**Table 3:** Clinical characteristics of patients operated by abdominoperineal excision in Sweden 2007–2009

		Total cohort, *n* = 1319			
		Included in the analysis	Deceased at follow-up	Non-responders	*p* value
Number of patients		545	467	307	
Sex	Female	218 (40.0 %)	170 (36.4 %)	142 (46.3 %)	0.076
	Male	327 (60.0 %)	297 (63.6 %)	165 (53.7 %)	
Age at operation		66.0	71.9	69.3	<0.001
BMI		29.3	27.3	26.8	0.944
ASA classification	ASA 1	144 (27.0 %)	69 (15.3 %)	61 (20.5 %)	<0.001
	ASA 2	314 (58.9 %)	235 52.2 %)	165 (55.4 %)	
	ASA 3	73 (13.7 %)	137 (30.4 %)	72 (24.2 %)	
	ASA 4	2 (0.4 %)	9 (2.0 %)	0	
Radiation therapy	None	64 (11.8 %)	123 (26.7 %)	64 (20.9 %)	<0.01
	Short (5 × 5 Gy)	355 (65.6 %)	222 (48.2 %)	181 (59.2 %)	
	Long (25 × 1,8/2 Gy)	122 (22.6 %)	116 (25.2 %)	61 (19.9 %)	
Chemoradiotherapy	Yes	110 (20.2 %)	112 (24.0 %)	57 (18.6 %)	0.560
	No	434 (79.8 %)	354 (76 %)	250 (81.4 %)	
Tumor height^a^		4,1	4,3	4,3	0.811
pT stage	T0–T2	256 (47.9 %)	98 (21.2 %)	130 (42.9 %)	0.385
	T3	252 (47.1 %)	289 (62.4 %)	156 (51.5 %)	
	T4	27 (5.0 %)	76 (16.4 %)	17 (5.6 %)	
pN stage	N0	344 (63.9 %)	187 (41.1 %)	202 (68.2 %)	0.354
	N1	130 (24.2 %)	117 (25.7 %)	67 (22.6 %)	
	N2	64 (11.9 %)	151 (33.2 %)	27 (9.1 %)	
Microscopic radicality^b^	Yes	522 (96.0 %)	386 (83.0 %)	285 (92.8 %)	<0.05
	No/indeterminate	22 (4.0 %)	79 (17.0 %)	22 (7.2 %)	
Perineal dissection	Conventional APE	71 (13.0 %)	79 (16.9 %)	59 (19.2 %)	<0.05
	ELAPE	222 (40.7 %)	172 (36.8 %)	124 (40.45)	
	Indeterminate	252 (46.2 %)	216 (46.3 %)	124 (40.4 %)	
Coccyx resection	Yes	124 (27.7 %)	133 (35.6 %)	73 (28.4 %)	0.850
	No	323 (72.3 %)	241 (64.4 %)	184 (71.6 %)	
Perineal reconstruction	Suture	430 (79.6 %)	352 (76.4 %)	238 (78.3 %)	0.803
	Mesh	64 (11.9 %)	56 (12.1 %)	36 (11.8 %)	
	Flap	46 (8.5 %)	53 (11.5 %)	30 (9.9 %)	

*p* values refer to differences between included patients and non-responders

*BMI* body mass index, *ASA* American Society of Anesthesiologists, *APE* abdominoperineal excision, *ELAPE* extralevator abdominoperineal excision

^a^Distance in centimeter from the lower edge of the tumor to the anal verge

^b^Circumferential resection margin >1 mm

The conventional APE and ELAPE groups differed regarding sex and tumor height, with more male patients and more distal tumors in the ELAPE group. The frequency of coccyx resection and method of perineal repair also differed between groups with very few resections and no use of flap or mesh in the conventional APE group. pT and pN stage and ASA grade were no different, and there were no significant differences regarding neoadjuvant radiotherapy and chemoradiotherapy (Table 4).

**Table 4:** Clinical characteristics of responders stratified on surgical technique

		Conventional APE	ELAPE	*p* value
Number of patients		71	222	
Sex	Female	38 (53.5 %)	87 (39.2 %)	<0.05
	Male	33 (46.5 %)	135 (60.8 %)	
Tumor height^a^		5,9	3.5	<0.001
Coccyx resection	Yes	2 (3.0 %)	95 (46.6 %)	<0.001
	No	64 (97.0 %)	109 (53.4 %)	
Perineal reconstruction:	Suture	71 (100.0 %)	120 (54.5 %)	<0.001
	Mesh	0	54 (24.5 %)	
	Flap	0	46 (20.9 %)	

Patients operated with an indeterminate surgical technique (*n* = 252) are omitted. Age, BMI, ASA grade, neoadjuvant treatment, pT and pN stage, and non-radical resections did not differ (data not shown)

*APE* abdominoperineal excision, *ELAPE* extralevator abdominoperineal excision

^a^Distance in centimeter from the lower edge of the tumor to the anal verge

### Perineal symptoms and associated distress

Perineal symptoms were present in 50 % of all responding patients and more frequently in women (58 vs. 44 %; *p* = 0.001). In total, 129 patients (24 %) experienced one or more perineal symptom of severe intensity, i.e., severe perineal morbidity according to our definition.

Tension between buttocks, sitting disability, cramps/urgency, and perineal pain were most frequently experienced (Table 5). Sitting disability was particularly distressful to patients. There was a positive relationship between the symptom intensity and the level of associated distress for all symptoms (data not shown).

**Table 5:** Perineal symptoms: prevalence, intensity, and associated distress

	Symptoms				Symptom-associated distress		
	None	Any intensity	Minor/severe^a^	Missing	None or low^b^	High^c^	Missing^d^
Pain	419 (79 %)	114 (21 %)	76 (14 %)/38 (7 %)	12	63 (62 %)	38 (38 %)	13
Sitting disability	410 (77 %)	123 (23 %)	81 (15 %)/42 (8 %)	12	57 (55 %)	47 (45 %)	19
Paraesthesia	461 (86 %)	76 (14 %)	53 (10 %)/23(4 %)	8	50 (68 %)	23 (32 %)	3
Tension	400 (75 %)	132 (25 %)	96 (18 %)/36 (7 %)	13	98 (75 %)	32 (25 %)	2
Tingling/stinging	490 (93 %)	40 (7 %)	33 (6 %)/7 (1 %)	15	28 (72 %)	11 (28 %)	1
Cramps/urgency	412 (77 %)	123 (23 %)	101 (19 %)/22 (4 %)	10	100 (82 %)	22 (18 %)	1

^a^Response options: *a little*/ *quite a bit* or *very much*

^b^Response options: *not at all* or *a little*

^c^Response options: *quite a bit* or *very much*

^d^Number of symptomatic patients who did not respond to the distress question

### Perineal wound healing

A total of 133 patients (25 %) reported delayed wound healing (>4 weeks) following their operation (Table 6). The wound healing process was associated with a high level of distress in 46 % of the patients. There was a positive relationship between the duration of the healing process and the level of associated distress (data not shown). Time to healing of the perineal wound was significantly related to surgical technique (Table 2). The relative risk of a delayed healing after ELAPE compared to conventional APE was 3.45 (95 % CI 1.46–8.15) after adjustment for smoking, diabetes, BMI, and preoperative radiotherapy. Radiotherapy alone was not associated with delayed healing in bivariate analysis.

**Table 6:** Time to healing of the perineal wound and patients’ perception of the healing process

			All	Missing
Time to healing (%)	Normal	0–4 weeks	391 (75 %)	21
	Delayed	1–4 months	91 (17 %)	
		>4 months	42 (8 %)	
Patients’ perception of the healing process		Not at all/a little difficult	284 (54 %)	21
		Quite/very difficult	240 (46 %)	

### Risk factors for perineal symptoms

Delayed perineal wound healing and female gender were statistically significant risk factors for severe perineal morbidity whereas no significant contribution could be observed for the other variables including radiotherapy (Table 7). In a subgroup of ELAPE patients, plain suturing and flap reconstruction both increased the risk of severe perineal morbidity compared with mesh repair. Resection of the coccyx did not have a significant impact (Table 8).

**Table 7:** Analysis of risk factors for *severe perineal morbidity* (corrected for age)

		Relative risk	95 % CI
Radiotherapy	Yes/no	0.816	0.455–1.463
Sex	Female/male	1.35	1.035–1.890
Perineal wound healing	Delayed/normal	1.546	1.132–2.111
Tumor height	>4 cm/0–4 cm	1.142	0.832–1.567

One or more perineal symptom of severe intensity (response options: *quite a bit* or *very much*)

*CI* confidence interval

**Table 8:** Subgroup analysis of risk factors for *severe perineal morbidity* in ELAPE patients (corrected for age)

		Relative risk	95 % CI
Perineal repair	Suture/mesh	2.311	1.023–5.222
	Flap/mesh	3.16	1.284–7.778
	Suture/flap	0.731	0.409–1.309
Excision of coccyx	Yes/no	1.078	0.440–2.640
Radiotherapy	Yes/no	1.484	0.727–3.028
Sex	Female/male	0.968	0.603–1.554
Perineal wound healing	Delayed/normal	1.624	1.001–2.636
Tumor height	>4 cm/0–4 cm	1.149	0.684–1.931

One or more perineal symptom of severe intensity (response options: *quite a bit* or *very much*)

*CI* confidence interval

### Perineal morbidity and quality of life

Patients who experienced one or more symptom of severe intensity (i.e., the group of 129 patients with severe perineal morbidity) had a significantly lower global quality of life as measured by EQ-5D VAS compared to those with less severe symptoms (median 75 vs. 83 points on the 100-point scale; *p* < 0.001).

## Discussion

This study showed that 50 % of patients had perineal symptoms 3 years after surgery. Tension between buttocks, sitting disability, perineal cramps/sensation of urgency, and perineal pain were most frequently experienced, and sitting disability was associated with most distress. A delayed perineal wound healing emerged as a risk factor for persistent perineal symptoms in our analysis. Delayed healing was considerably more common after ELAPE compared to conventional APE with more than three times higher risk in ELAPE patients. Notably, this did not translate into more long-term perineal symptoms in ELAPE patients (data not shown). Finally, the experience of one or more perineal symptom of severe intensity was associated with a significantly lower global quality of life score in the EQ-5D visual analogue scale.

Several studies have reported high rates of short-term perineal wound complications after abdominoperineal excision in general and ELAPE in particular [11, 24], but few have explored long-term perineal morbidity in these patients. Musters et al. [11] found a perineal hernia or a persisting perineal sinus in 8–10 % of patients about 2 years after any kind of abdominoperineal excision. Welsch and colleagues [23] reported a frequency of persistent pain or other (unspecified) functional deficits of 50 % after ELAPE about 2 years after surgery. However, these studies are limited by small sample sizes, varying follow-up times, and lack of detail regarding perineal symptoms.

The present study is large in comparison to most other studies. It combines data from three different relevant sources. Included patients were collected from a prospective national registry with almost complete coverage, which strengthens the external validity. Original operative notes were retrieved and systematically analyzed, and finally, we used data on patients’ self-reported symptoms at a defined time point after surgery. In this way, we avoided the pitfalls of retrieving information on symptoms from hospital records, with their inherent bias and inconsistency of symptom documentation. This enables us to draw reliable conclusions.

We did not attempt to design an instrument to reflect perineal morbidity in a single score; instead, questions were analyzed individually. Patients were queried not only about the intensity of symptoms but also about the perceived significance (symptom-associated distress) of symptoms. Although not a new concept in oncologic and epidemiologic research [21, 22], it has seldom been studied in patients with rectal cancer before.

We have used the term severe perineal morbidity, defined as the experience of at least one symptom of severe intensity, in order to identify patients with more severe perineal symptoms. This definition is arguably arbitrary but it is clinically reasonable in our opinion.

Healing of the perineal wound was defined as *delayed* if the process exceeded 1 month. Wound healing is difficult to study; once patients have been discharged, outpatient visits are generally few and wound care is often carried out in a primary care setting. In our study, time to healing of the perineal wound was a patient-reported variable. We asked about the period of time during which the patient needed wound care after the operation, either at home, in a hospital, or at a primary care facility. This should correspond well with the time it took for the wound to heal. Although there is a risk of recall bias regarding this outcome, we believe that patients, even after 3 years, are able to give a fair estimate of the duration of the healing process, as it is often distressful. Notably, healing time did not differ significantly between patients receiving radiotherapy short course, long course, or not at all (data not shown).

The differences between responders and non-responders presented in Table 3 should be considered in relation to the generalizability of results. Non-responders were older with more comorbidity and fewer received radiotherapy compared with responders. However, whether these differences lead to overestimation or underestimation of perineal morbidity in the total population of 3-year survivors is unclear.

Although there are missing data for most variables and questions, numbers are low and should not disturb interpretation.

The retrospective non-randomized cross-sectional design has limitations. Baseline data on perineal symptoms are not available. However, this is probably not a major concern as perineal symptoms before treatment are likely to be infrequent. An alternative study design would be a prospective observational study. We are currently running such a study in 16 centers in Sweden and Denmark, but as inclusion is still ongoing, long-term follow-up results are not available [3].

For the long-term management of patients treated for distal rectal cancer, solid data on functional impairments are valuable. We present such data regarding perineal symptoms based on a national cancer registry for the first time. We conclude that the prevalence of persistent perineal symptoms is high following abdominoperineal excision. This should be conveyed to patients as part of the preoperative counselling along with information about other potential functional consequences of the procedure. Patients would probably benefit from closer surveillance and support during the wound healing process.
